# Work related injuries in Qatar: a framework for prevention and control

**DOI:** 10.1186/s12995-018-0211-z

**Published:** 2018-09-05

**Authors:** Amber Mehmood, Zaw Maung, Rafael J. Consunji, Ayman El-Menyar, Ruben Peralta, Hassan Al-Thani, Adnan A. Hyder

**Affiliations:** 1 Johns Hopkins International Injury Research Unit, Health Systems Program, Department of International Health, Johns Hopkins Bloomberg School of Public Health, 615 N Wolfe St, Baltimore, MD 21205 USA; 2 HMC Injury Prevention Program, Hamad General Hospital, Hamad Medical Corporation, Doha, Qatar; 3 Trauma Surgery Section, Hamad General Hospital, Hamad Medical Corporation, and Weill Cornell Medical College, Doha, Qatar; 4 Universidad Nacional Pedro Henriquez Urena (UNPHU), Santo Domingo, Dominican Republic; 5 Department of Surgery, Hamad General Hospital, Hamad Medical Corporation, Doha, Qatar; 6 Johns Hopkins Berman Institute of Bioethics, Baltimore, MD USA; 7 George Washington University Milken Institute School of Public Health, Washington, DC USA; 8 Weill Cornell Medical College, Doha, Qatar

**Keywords:** Work-related injuries, Occupational injuries, Qatar, Injury prevention, Migrant workers, Middle East, Labor migration

## Abstract

Work related injuries (WRIs) are a growing public health concern that remains under-recognized, inadequately addressed and largely unmeasured in low and middle-income countries (LMIC’s). However, even in high-income countries, such as those in Gulf Cooperating Council (GCC) like Qatar, there are challenges in assuring the health and safety of its labor population. Countries in the GCC have been rapidly developing as a result of the economic boom from the petrochemical industry during the early seventies. Economic prosperity has propelled the migration of workers from less developed countries to make up for the human resource deficiency to develop its infrastructure, service and hospitality industries. Although these countries have gradually made huge gains in health, economy and human development index, including improvements in life expectancy, education, and standard of living, there remains a high incidence of work-related injuries especially in jobs in the construction and petrochemical sector. Currently, there is scarcity of literature on work-related injuries, especially empirical studies documenting the burden, characteristics and risk factors of work injuries and the work injured population, which includes large numbers of migrant workers in many GCC countries. This paper will focus on the current understanding of WRIs in those countries and identify the gaps in current approaches to workplace injury prevention, outlining current status of WRI prevention efforts in Qatar, and propose a framework of concerted action by multi-sectoral engagement.

## Background

Work-related (or occupational) injuries (WRI) are a significant cause of death worldwide, whereas non-fatal WRIs result in long-term disability or prolonged leave from work [1]. WRI is defined by the International Labor Organization (ILO) as “an unanticipated and unplanned occurrence including acts of violence resulting from and in connection with work which cause one or more workers to incur a personal injury, disease or death” [2]. The Occupational Safety and Health Administration of the United States, also deems an injury to be work-related if an event or exposure in the work environment either caused, or contributed to, the resulting condition or significantly aggravated a pre-existing injury [3].

WRI result not only in fatalities and disabilities, but also leads to decreased productivity from lost work-days and loss of skilled workers. The average economic costs of work-related illnesses and injuries is 4% of gross domestic product (GDP) but varies between countries with an estimated 1.8% to 6% in places such as United States, Australia, and Singapore [4]. According to the ILO, 7600 people die every day as a result of work-related injuries or illnesses, with 15% of deaths directly attributable to WRI [1, 5]. About 6 of every 1000 workers will be fatally injured on the job during a 40 year work span in the United States [6]. Despite underreporting, the Global Estimates of Occupational Accidents and Work-related Illnesses (2014) reported that approximately 289 out of 313 million (92%) cases of WRIs occurred in low- and middle-income countries [5, 7].

According to the ILO, certain occupations and industries are known to have a higher risk for WRIs because the nature of the job and the conditions where the work is performed increase the risk of injuries or exposure to hazardous agents. In high income countries, the rate of WRI has dramatically declined due to investment in interventions focusing on hazard mitigation, occupational safety and health. In the United States, work-related fatalities between 1933 and 1997 decreased from 37 to four per 100,000 workers, and work-related road traffic injuries declined from 18 to 1.7 per 100 million vehicle miles traveled. These successes were dubbed as two of the top ten leading public health achievements in the United States [6, 8].

Some countries in the Middle East, such as members of the Gulf Cooperating Council (GCC) have rapidly developing economies after the boom in the oil industry during the early 1970s and have adopted a policy of hiring an expatriate labor force to make up for their human resource deficiency to support the infrastructure, service and hospitality businesses. Although these countries have gradually made considerable gains in health, economy and human development index (Table 1) [9], there is a concern about high incidence of WRIs especially in construction and oil-industry related jobs, and related economic losses [9–11]. Currently there is scarcity of literature on WRIs, especially empirical studies documenting the burden, characteristics and risk factors for WRIs and the work injured population, which includes large numbers of migrant workers in many GCC countries like Qatar [10–12].

**Table 1:** Human development indicators for Gulf Cooperating Council countries, 2016

	Human development Index (HDI)^a^	Average HDI growth (%) 1990–2015	GDP per capita PPP $ (2015)^c^	Life expectancy at birth (years)	Expected years of schooling	Under-five mortality rate (per 1000 live births)
Bahrain	0.824	40	44,182	76.7	14.5	6.2
Kuwait	0.8	46	67,113	74.5	13.3	8.6
Oman	0.796	121^b^	35, 983	77	13.7	11.6
Qatar	0.856	51	135,322	78.3	13.4	8
Saudi Arabia	0.847	77	50,284	74.4	16.1	14.5
United Arab Emirates	0.84	58	66,102	77.1	13.3	6.8

^a^Human Development Index integrates life expectancy at birth, mean years of schooling and gross national income per capita

^b^Data available from 2000 to 2015 for Oman

^c^Gross domestic Product (GDP) estimated using the purchasing power parity (PPP)

This paper will focus on the current understanding of WRIs in Gulf states, specifically Qatar, and identify the gaps in current approaches to WRI prevention, outlining the current status of WRI prevention efforts in Qatar; and propose a framework of concerted action through multi-sectoral engagement.

## The burden of WRIs in the GCC and neighboring countries

Economic prosperity and job opportunities in the middle east, particulary in gulf countries where the demand for hydrocarbon products has fueled economic growth and, along with it, infrastructure development has propelled the migration of workers from LMICs [13]. In a study published in 2006 from Turkey, Egypt, Morocco, and Tunisia, the fatal occupational “accident” rate was estimated to be 21.2 per 100,000 employees in agriculture, 21.2 per 100,000 in industry, and 12.4 per 100,000 employees in service [4]. While in Jordan, the fatality rate was estimated to be 25.5 per 100,000 [14].

In a recent study from Oman, the injury rate amongst oil field workers was reported to be 1980 per 100,000 [11], and the mortality rate of WRIs in the United Arab Emirates was estimated to be 136 per 100,000 workers per year in 2009, where unintentional injuries are the second leading cause of death among the expatriate population and 21% of all non-fatal injuries were a result of WRIs [10, 15–17]. Morbidity and mortality related to WRIs and other illnesses among migrant workers are disproportionately higher when compared to native workers in the GCC [18]. With the lack of proper surveillance systems, these numbers are likely to be gross underestimates of the true number of WRIs and their consequences. Therefore, not only WRIs are a major public health issue in this region, but also affecting a large vulnerable population.

### Qatar: Current status of labor force and WRIs

**Fig. 1:**
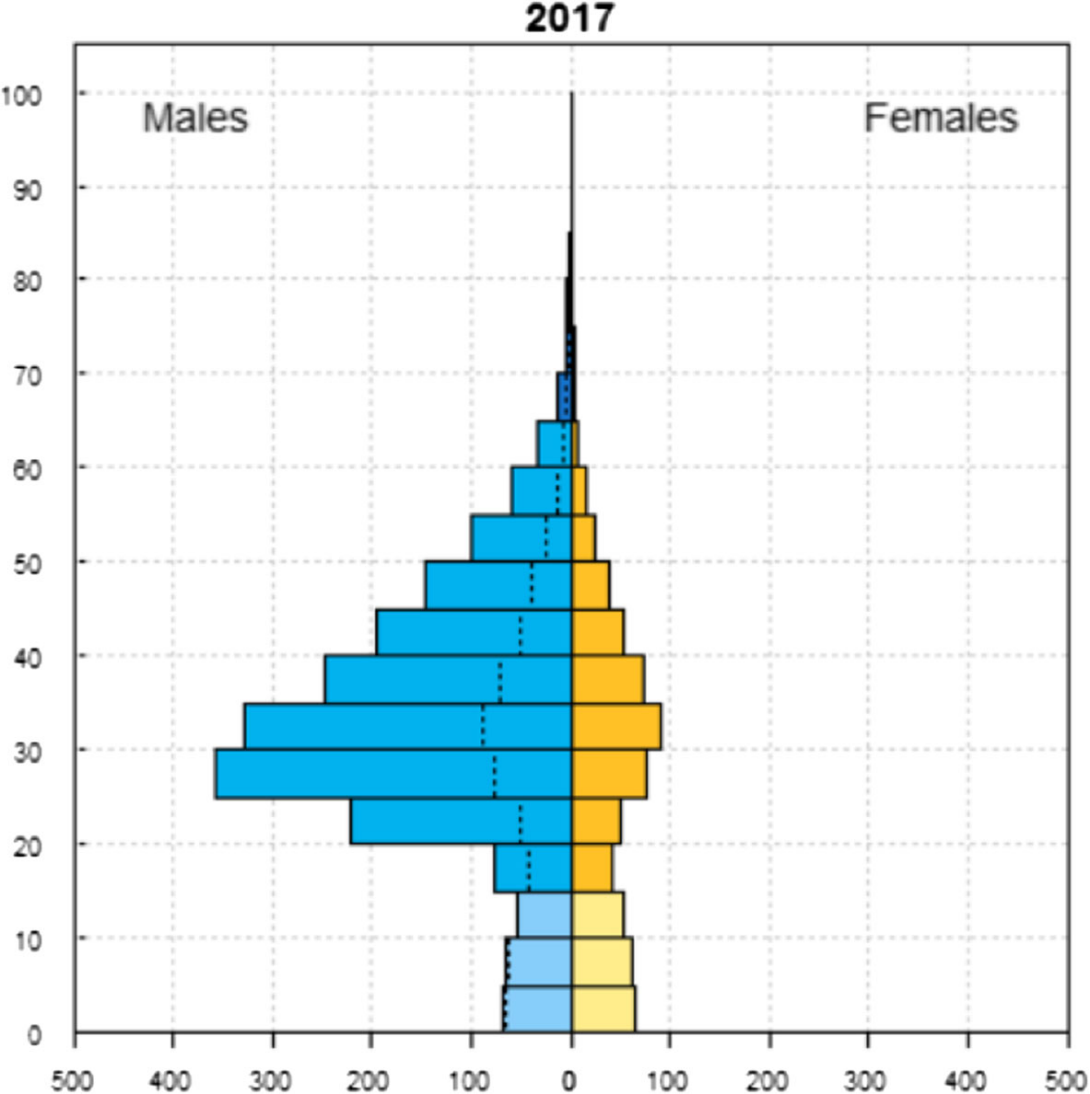
Qatar 2017 demographic profile. Source: United Nations, Department of Economic and Social Affairs, Population Division (2017). World Population Prospects: The 2017 Revision (On X-Axis, Numbers expressed in × 1000 for each age group; on Y- axis: age in years. The dotted line indicates the excess male or female population in certain age groups)

Qatar is a rapidly developing, oil rich, small gulf country within GCC with a total population of 2,569,804 in 2016 [19, 20]. Development across different sectors has attracted a large migrant or “expatriate” (expat) worker population (Fig. 1), who are now employed in diverse industries; from highways, rail networks, seaports, airports, oil facilities, chemical factories, residential and commercial facilities to the construction of stadiums [21].

Qatar has witnessed rapid population growth from just 373,392 in 1986, which stems largely from the influx of migrant workers that is estimated to make up approximately 85.7% of population, or 94.1% of the employed population in 2013 [20, 22]. Expat workers are largely of South Asian origin with other nationalities such as Filipino and Egyptians also making modest contributions [23]. Within this foreign labor population, men exceeded women by a ratio of 8 to 1 (Fig. 1). The largest proportion (39.2%) were employed in the construction industry, primarily to fulfill the requirements of rapid infrastructure development for the much anticipated 2022 FIFA World Cup [20]. Ensuring the health and safety of the working migrant population is challenging since the majority work in what is known as the “3D” sector - the dangerous, dirty, and difficult jobs [24].

Most current information on WRIs in Qatar is based on hospital-based studies capturing moderate to severe injuries (Table 2). A study based on trauma registry data in Qatar reported that a significant proportion of severe WRI affected construction workers (42%) [25]. Fall from height was the major contributor for WRIs [12]. Another study reported that the incidence of fall injuries for a period of one year was 86.7 per 100,000 workers with fatality rate of 8.44 per 100,000 workers. The annual cost of providing care to these patients was estimated to be over 4.4 million USD, with a mean cost of $15,735 per patient [26].

**Table 2:** Selected studies describing epidemiology of WRIs in Qatar

Author and year	Title of the study	Major finding
Consunji et al. 2017 [54]	Epidemiologic and temporal trends of work-related injuries in expatriate workers in a high-income rapidly developing country: Evidence for preventive programs.	Although there was a 37% reduction of the incidence of injury per 100,000 workers, from 2008 to 16, the proportion of falls from height decreased and that from RTIs increased.
Al-Thani et al. 2015 [12]	Epidemiology of occupational injuries by nationality in Qatar: Evidence for focused occupational safety programmes	Most of the workers experiencing WRIs were from Nepal (28%), India (20%) and Bangladesh (9%). Fatal WRIs were predominately among Indians (20%), Nepalese (19%), and Filipinos/Bangladeshis (both 8%)
Al-Thani et al. 2014 [25]	Workplace-Related Traumatic Injuries: Insights from a Rapidly Developing Middle Eastern Country	WRI patients are mainly laborers involved in industrial work (43%), transportation (18%), installation/repair (12%), carpentry (9%), and housekeeping (3%). A vast majority of workers (64%) did not use protective devices
Tuma et al. 2013 [26]	Epidemiology of workplace-related fall from height and cost of trauma care in Qatar	Incidence of fall related WRI was 86.7 per 100,000 and associated death rate was 8.44 per 100,000 workers.
Bener et al. 2011. [41]	Trends and characteristics of head and neck injury from falls: A hospital based study, Qatar	Among 1952 patients who were treated at a major trauma center for head and neck injuries, nearly half of them suffered from falls during work
Bener et al. 2012 [34]	Trends and characteristics of injuries in the State of Qatar: hospital-based study	This 5-year study demonstrated that overwhelming majority were non-Qatari males and over 50% of 46,701 injuries were related to WRIs. Common injuries included injuries of head and neck, extremities, and back.
Khan et al. 2005 [30]	Study of Patients with Heat Stroke Admitted to the Intensive Care Unit of Hamad General Hospital, Doha, Qatar During Summer 2004.	This case series highlighted the WRIs resulting from heat stroke and its medical complications during the hot summer months

The case fatality rate for WRIs in Qatar is much higher when compared to other high-income countries like the United Kingdom and United States. Many international organizations have voiced concerns about construction workers exposed to other risks such as high temperature, humidity, noise and long works hours, some of which have been documented previously in scientific papers [27–30]. These concerns have served as an impetus for the government to follow these recommendations, in order to minimize the risks to worker health and take steps towards a safer work environment, mandated by tough policies and robust monitoring systems [31].

The incidence of WRIs in Qatar is similar to rates seen in other low- and middle-income countries and partly attributable to lack of a cohesive occupational health and safety infrastructure, as well as regulations and enforcement of policies [18]. There is limited data available in the GCC as to whether identified risk factors and proven mitigation strategies from other high-income countries were applicable in the local context [32]. There are additional challenges in GCC countries for occupational health and safety that largely stem from the culturally, economically, ethnically, and socially heterogeneous nature of the worker population. More focused research is needed to better understand the nature of WRIs, and understanding of workers’ skills and training, job experience, use of protective equipment and risk perceptions [33].

Employing large numbers of migrant workers could also stress the existing healthcare infrastructure. In a study published in 2012 from Qatar, a high burden of WRIs on their health system was reported. Of 53,366 patients visiting the hospital over year period, 88% were migrant workers and road traffic injuries, occupational falls and construction work were among the top three causes of WRIs [34].

## A model approach to address WRI burden

**Fig. 2:**
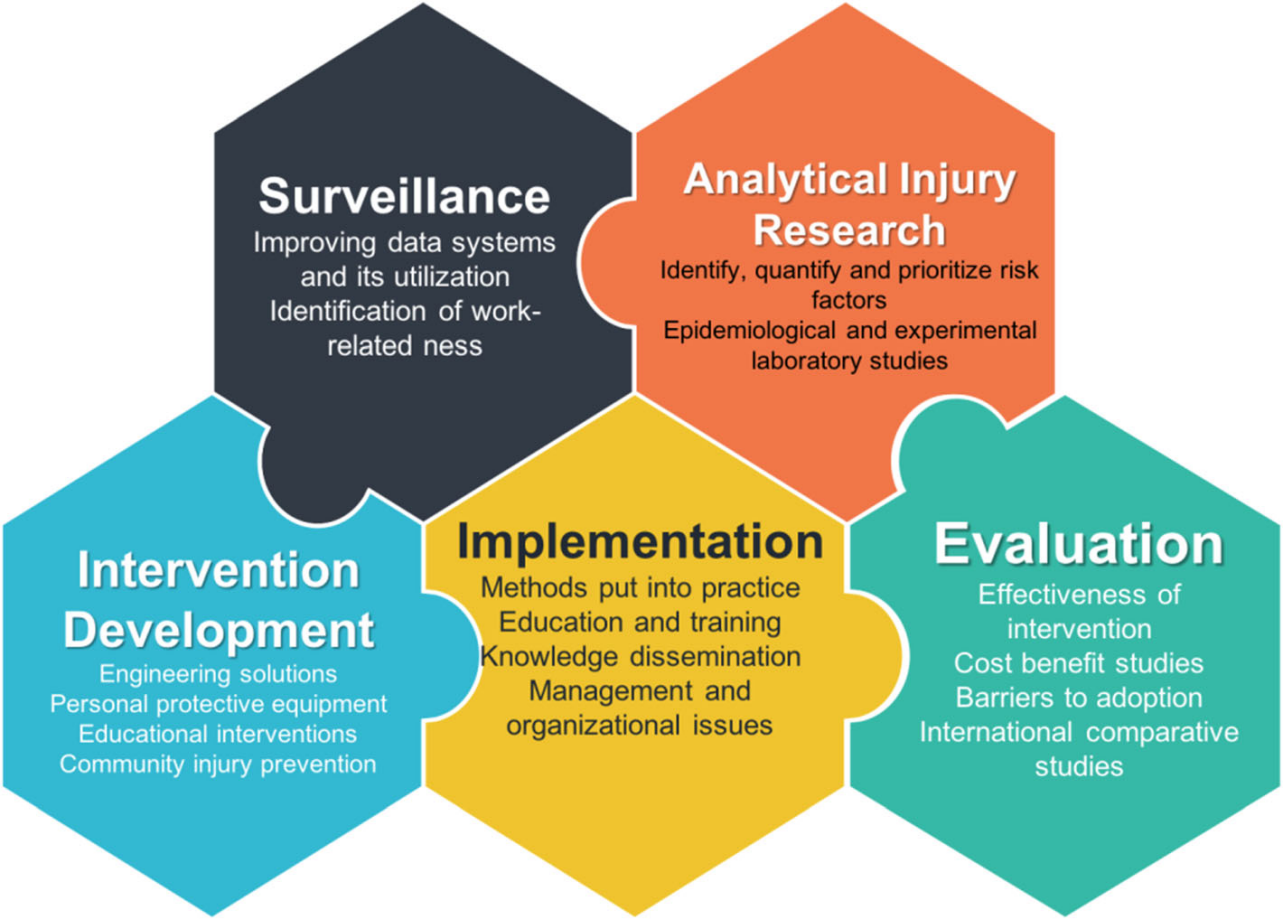
The public health approach to occupational injury prevention. Adopted from G. S. Smith – Public health approaches to occupational injury prevention: do they work?[6]

The most promising strategy for WRIs is a public health approach that incorporates policy and research into practice, interventions, and training. Such a process would have its foundations in collecting up-to-date and credible data on workplace hazards, environment and workers’ health policies, assessment of risks, education for employers and workers, participation in prevention campaigns, and referral to necessary services [35]. A public health framework (Fig. 2) often utilized in injury prevention could be adapted for WRI control and prevention [6]. This framework builds upon the relationship of problem identification, analytical injury research to facilitate development and implementation of strategies, and continuous monitoring and evaluation of interventions.

The most successful economies have demonstrated that workplaces designed according to the principles of occupational health, safety and ergonomics are also the most sustainable and productive [36, 37]. At the core of these principles, lies the foundation of all effective public health strategies and interventions: good data that can be used to develop, implement and monitor evidence-based policies, specific to the community. Active surveillance systems provide up-to-date information on WRI’s, this could be achieved through the development of a unified WRI database to quantify the WRI burden and identification of risk factors and work place hazards.

Risk factor identification and hazard mitigation is facilitated by government’s policies that address workers’ protection, environmental safety, and maintaining safety standards in industrial and domestic sectors. To put the methods in practice, education and training of the labor force is integral, combined with guidelines and supervision to promote safe behavior at work tailored to the needs of workers’ population. Engagement, encouragement, and incentivization of different stakeholders including representatives of labor work force to integrate and expand injury prevention activities must be prioritized.

**Fig. 3:**
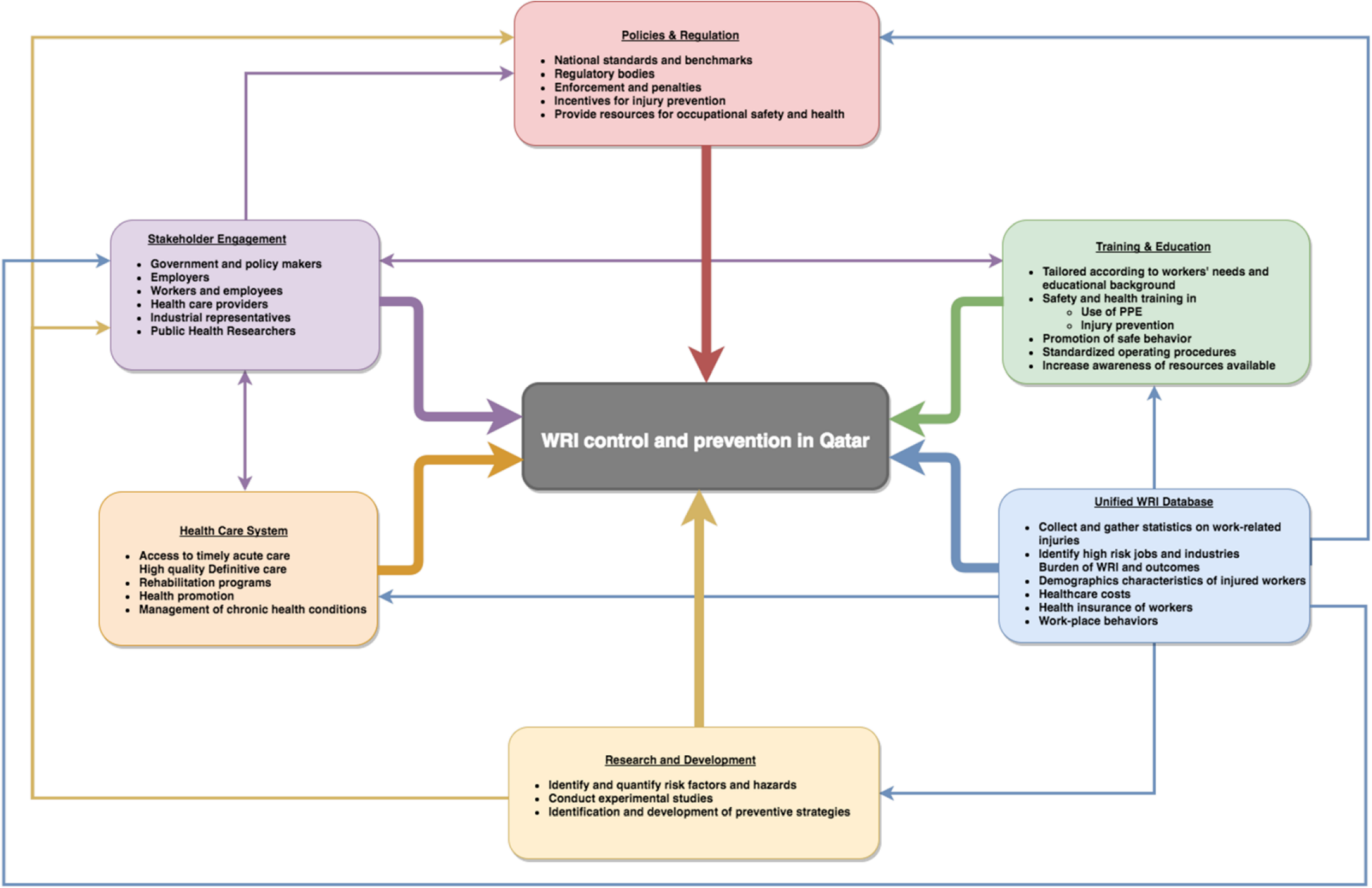
A comprehensive framework for WRI prevention and control in Qatar. Adopted from WHO Injury Surveillance Guidelines [38]

Based on the injury prevention framework outlined above, we propose a comprehensive framework integrating WRI prevention and care for injured workers in Qatar (Fig. 3), that is largely based on World Health Organization injury surveillance guidelines [38]. This framework incorporates the principles of injury control into the context of Qatar and builds upon the seminal work already undertaken to improved work place safety and systematic reforms to promote injury control by different organizations. Each entity (stakeholders, health system, research and development) is linked with others through a common path of active surveillance and information sharing.

### Identification and engagement of stakeholders

WRI prevention and control involves a multitude of players and stakeholders. Identifying and engaging with important players, and using a participatory approach to policy development and implementation is the key to ensure buy-in and collaboration, and may necessitate the creation of a dedicated forum or high-level committee. Government officials, ministry representatives, industrial representatives, contractors and employers provide support and resources for implementation. Epidemiological knowledge, international safe practices, evidence based strategies, advocacy, and monitoring the impact of policies and interventions are best represented by the academics, employees, health professionals, legal experts, and international organizations.

### Health informatics: Unified database on occupational injuries

Effective injury prevention is contingent on the development of good data systems. A unified database of injuries, where every entity that employs workers is mandated to submit details of incidents and injuries in a standard format, could potentially create the most robust platform of WRI surveillance. Existing data are often not collected for this purpose, often not shared and thus different databases are disconnected and frequently remain uncoordinated. WRI surveillance would require data input across different sectors including ministries of public health, labor, social affairs, etc., private and government health care providers, small and large companies, and corporations. Regular data analysis and dissemination of findings to all stakeholders will help develop evidence-based policies, set research priorities, agenda for training and education and monitor the cost implications of WRI burden.

### Laws, policies, standards, and regulatory bodies

A comprehensive policy framework for occupational health and safety is needed to support education, training, research and healthcare services to prevent WRIs. A central regulatory body could facilitate the implementation and monitoring of these policies, ensuring adherence with international standards of safety. Judicial use of incentives by government could expedite the uptake of policies and interventions by different sectors and improve adherence with international safety standards.

Introduction of on-site health teams, inspections of safety practices, mandatory reporting of adverse incidents and injuries could promote organizational safety culture. Empowering and training the workers to be on-site inspectors will bolster the power and impact of regulatory bodies through a bottom up approach [39]. They can also be used to address common complaints, claims and concerns of the employees, or refer them to higher authorities through well-defined channels.

Efforts could also be directed towards strengthening bilateral treaties with the migrant workers’ states of origin. Frameworks of official communication and information sharing could be streamlined through clear policies, and redress processes should be made easier and transparent. Workers’ access to policies, procedures, and official documents produced by relevant ministries must be ensured, along with improved interpretation and translation services.

### Research and Development

The epidemiology of injuries in Qatar demonstrate a higher burden of WRIs in construction jobs, falls and RTIs. Research addressing hazard identification in construction sector, research to mitigate risk of fall related WRIs, and identification of modifiable injury risk factors is needed. Ergonomic interventions, environmental protection, and human resource management are only some of a long list of broad research topics that could provide essential and contextual information on how to protect health and improve safety without compromising productivity in the context of Qatar [36, 37, 40]. As reported previously, many WRIs result in head and neck, extremity and back injuries (Table 2) [41]. Short- and long- term consequences of these injuries including cumulative economic cost of treatment, loss of productivity, rehabilitation and replacement have not been studied. Priority should be placed on improving health outcomes, prevention of disability, psychological and mental health impact of WRIs among workers who frequently live without family support [42]. Cost benefit analyses could help determine effectiveness of WRI control strategies vs. cost of treatment, decreased productivity from lost work-days, and repatriation.

### Education and training of the workforce

The hierarchy of hazard control is often utilized across industries to minimize or eliminate exposure to workplace hazards [43]. Personal protective equipment is at the bottom of the hierarchy and is the least effective measure of hazard control but is also the minimum protection provided to all workers. Workers should receive the education and training for identifying workplace hazards and provided with appropriate equipment and training for its operation and/or use.

Qatar where the majority of the workforce are from different backgrounds, may show variability in knowledge and behavior about workplace safey. Other countries with the same issue have developed and incorporated educational and training materials for safety and health targeting migrant workers [44, 45]. Education and training programs would require specific customizations that would increase comprehension of material to the migrant population. This may necessitate offering programs that are linguistically and culturally appropriate, in languages that are understood and spoken fluently by the workers.

### Healthcare system: Access to care and rehabilitation

Injured workers seek medical care at different points throughout the healthcare infrastructure depending on the severity of the injury. Medical care provided at the trauma centers is likely to reflect more severe injuries; for workers with less severe injuries, treatment may be sought at primary care facilities, through private healthcare providers, or they may not receive any care at all. Recent studies have shown that WRIs are a leading cause of hospital visits in Qatar; less is known about the burden and outcome outside of hospitals [41]. Currently, all expatriates and migrant workers are provided with a heath card that provides access to all public hospitals and clinics. Additionally, the Qatar Red Crescent has been providing therapeutic services to workers for some years. However, accessing acute care in a timely manner remains difficult for many migrant workers [46]. The barriers to access care may include fear of losing job or part of the wage, inadequate knowledge of the use of health card, long travel to reach the tertiary care medical center, which may be undesirable for employer, or puts the employee at the peril of using extra resources, such as service fee and transportion charges. [47].

## State of policy environment for WRIs in Qatar

The legislative and regulatory framework for work-related injuries is still developing in Qatar [48]. The current legislations governing workplace injuries are laid out in the Qatar Labor Laws [31]. Qatari labor law requires employers to inform their workers of hazards associated with the work and safety precautions that should be exercised, to protect the workers from injuries, disease and accident, provide personal protective equipment and gear, hygiene and good ventilation, first aid box and periodical medical checkup [49]. Failure to comply with these procedures or violation of standard safety measures may result in possible closure of the work site, or fine or imprisonment or both. A Decree by the Ministry of Civil Service and Housing Affair prohibits working on areas exposed to the sun between 1130 and 1500 h during the hottest months of the year. Although this seems very promising in ensuring workers’ welfare, the implementation of these laws has not been effective or uniform across sectors [29]. With recent reforms in the Qatari labor laws, the government has taken steps to further strengthen policies to protect the rights of domestic workers [50].

The National Occupational Health and Safety Committee was established in 2011 under the Ministry of Labor and Social Affairs, and the Supreme Council of Health, as part of the National Health Strategy to improve governance and regulation of WRIs. Current legislation does dictate that the employer assume responsibility for medical expenses and salary for work-related injuries. Despite the existence of such legislations and regulations, many migrant workers may not be aware of it, which calls for better dissemination of information. Trade unions or committees for foreign workers are prohibited in Qatar and the Qatari government only allows Qatari workers to “to strike, form committees, and join international labor organizations, pending ministerial approval” [51].

Recently, the Qatar Red Crescent in agreement with the Ministry of Public Health [formerly the Supreme Council of Health] established a health center dedicated to expatriate health care needs, with a capacity to receive 32,000 visitors per month [52]. So far, there is one government state-of-the-art rehabilitation institution operating in Qatar, which started functioning in 2016 to offer five rehabilitation programs [53]. It is unclear at this point, if the services would be expanded to include occupational therapy and rehabilitation for WRIs.

Under the Qatar Foundation National Priority Research Programs, a research project was launched in 2015 to initiate and implement a targeted unified workplace injury surveillance system to inform policies and programs to reduce the health burden, and the healthcare costs from WRI’s in Qatar. Under this collaborative project, a stakeholder network was established to discuss the WRI problem, engage and exchange ideas, prioritize research agenda, as well as push the efforts towards identification, evaluation, and integration of WRI data sources. New efforts are currently under way at Red Crescent and Hamad Medical Center to place an electronic tag in medical records, on all patients attending their outpatient and inpatient facilities with WRI. Additionally, empirical data on risk factors and vulnerable populations is being collected and analysis of health care data to measure the burden, risk factors, hospital outcome and cost of treatment has been conducted [54].

## Discussion

Work-related injuries are debilitating not only to the injured worker, but also to the country’s productivity and economy. Despite economic growth, GCC nations like Qatar, have their own challenges in assuring the health and safety of its migrant worker population. Currently, there is a dearth of data from Qatar and other neighboring states, with media reports of increased incidence of WRIs in migrant workers [27, 28]. WRI related information is fragmented among different industries, governmental bodies, healthcare system and is collected mainly for legal or care related documentation. A concerted effort by all stakeholders, guided by evidence collected through a unified database registry, that gives a strategic direction to preventive efforts, effective interventions and evaluation of impact of different policies will steer the country towards the common goal of WRI prevention and control.

While there are well established safety practices that should be adopted in all work environments, some risk factors are variable across different industries, dependent on individual worker characteristics, and affected by workplace organizational policies and practices [55–58]. The most recent example is from Chile, where Fatal Work Accidents Registry captured work related mortality, high risk occupations, vulnerable population and characteristics of industry with high fatality risk [59]. Thus, blind adoption of strategies to prevent workplace injuries may not be effective, could be costly and such efforts may fail or be inadvertently harmful. Some of the core principles incorporated in the policies of the countries with the strongest occupational health and safety traditions include primary prevention and use of safe technology; governance and stewardship to monitor and regulate working conditions. Integration of production and healthy activities enhances employees’ own interest in health and safety at work.

Given a complex environment and upkeep of WRI prevention agenda, priorities for Qatar include establishing and enforcing legislation that protects the health of workers in the construction industry. Using our proposed framework (Fig. 3), WRI control and prevention can be achieved through a multi-strategy approach that (1) empowers employers and workers to share responsibility for the safety and health of all; (2) acknowledges that government have the authority and responsibility to develop and roll out appropriate policies and create and an equitable environment that places workers safety and health on priority; (3) highlights the importance of research and development to advance the agenda of evidence informed policy making; (4) recognizes that training and education of employees in a multicultural and multilinguistic context, especially unskilled labor workforce requires customization; (5) places a priority on health care needs of the WRI patients, both in acute phase and during rehabilitation and finally (6) the foundation of this framework lies on a good and up-to-date WRI surveillance systems that provides necessary input for policy, training, and monitoring and evaluation of interventions to control WRI. This approach could become the pioneering effort within Qatar and an example for the neighboring countries with a similar economic environment and rapidly changing labor force.

## Abbreviations

GCC: Gulf Cooperating Council
GDP: Gross Domestic Product
ILO: International Labor Organization
WRI: Work-related injuries
